# Platelet Disorders and Medication Strategies

**DOI:** 10.1055/a-2561-8818

**Published:** 2025-05-07

**Authors:** Zhao Zhang, Xianghui Zhou, Xiyuan Fang, Xin Zhou, Zhipeng Cheng, Yu Hu

**Affiliations:** 1 Department of Hematology, Union Hospital, Tongji Medical College, Huazhong University of Science and Technology, Wuhan, People's Republic of China; 2 Department of Stomatology, Union Hospital, Tongji Medical College, Huazhong University of Science and Technology, Wuhan, People's Republic of China

**Keywords:** platelets, thrombocytopenia, thrombocytosis, medication

## Abstract

Platelets are among the most abundant cells in the body and play important roles in coagulation and immunity. Platelets are formed when hematopoietic stem cells proliferate and differentiate into megakaryocytes via the regulation of various cytokines. After the megakaryocytes mature in the bone marrow cavity, proplatelets are released into the blood circulation where they eventually remodel into mature platelets. Given that the production and functions of platelets involve the regulation of many factors—such as hematopoietic stem cells, the hematopoietic microenvironment, and cytokines—the causes and mechanisms of platelet-related diseases are diverse, often involving platelet production, clearance, and distribution. In this review, we examined the regulation of platelet production and summarized common disorders affecting platelet quantity, namely, thrombocytopenia and thrombocytosis. In addition, we reviewed previous clinical studies and summarized the medication strategies commonly used for the treatment of different platelet disorders in different clinical scenarios.

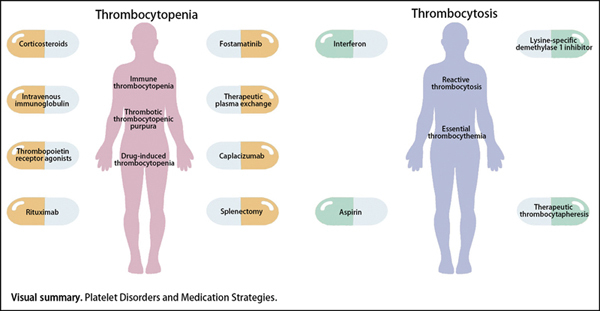

## Introduction

Platelets play important roles in hemostasis and immune response. 1 2 Platelets are among the most abundant cells in the human body and are mainly derived from bone marrow megakaryocytes (MKs); however, the results of some studies suggest that some platelets are produced in the lungs. Platelet production involves several stages that can be roughly divided into the hematopoietic stem cell, macrophage, proplatelet, and mature platelet stages. Each stage is regulated by several factors, and dysfunction of any regulatory factor may affect platelet production.

Platelet disorders can be divided into two categories: thrombocytopenia and thrombocytosis. Thrombocytopenia is typically caused by decreased platelet production (such as in cases of bone marrow suppression after chemotherapy and MK maturation disorder), increased platelet destruction (caused by conditions such as production of antiplatelet antibodies and hypercoagulability), and abnormal platelet distribution (such as in cases of hypersplenism and splenomegaly) ( Table 1 ). 3 The most common causes of thrombocytosis are reactive thrombocytosis and myeloproliferative neoplasms. In this review, we summarized the common causes of thrombocytopenia and thrombocytosis starting from the regulation of thrombopoiesis, and reviewed the common medication strategies used for the management of both disorders. We believe that this review can guide clinical treatment and promote the conceptualization and conduction of future research on platelet disorders.

**Table 1:** Etiologies of thrombocytopenia

Decreased platelet production	Increased platelet destruction		Abnormal platelet distribution	Other
	Immune-related	Non-immune-related		
Bone marrow failure, suppression, or infiltration	Immune thrombocytopenia	Thrombotic microangiopathy (thrombocytopenic purpura, hemolytic uremic syndrome, drug-induced thrombotic microangiopathy)	Hypersplenism	Pregnancy with thrombocytopenia
Chemotherapy or radiotherapy	Drug-induced immune thrombocytopenia	Disseminated intravascular coagulation	Dilutional thrombocytopenia	EDTA-dependent pseudothrombocytopenia
Nutritional deficiencies	Paroxysmal nocturnal hemoglobinuria	Mechanical damage		Wiskott-Aldrich syndrome
Infections				Bernard-Soulier syndrome
Congenital amegakaryocytic thrombocytopenia				Alcohol or toxins

## Blood Platelet Formation

**Fig. 1:**
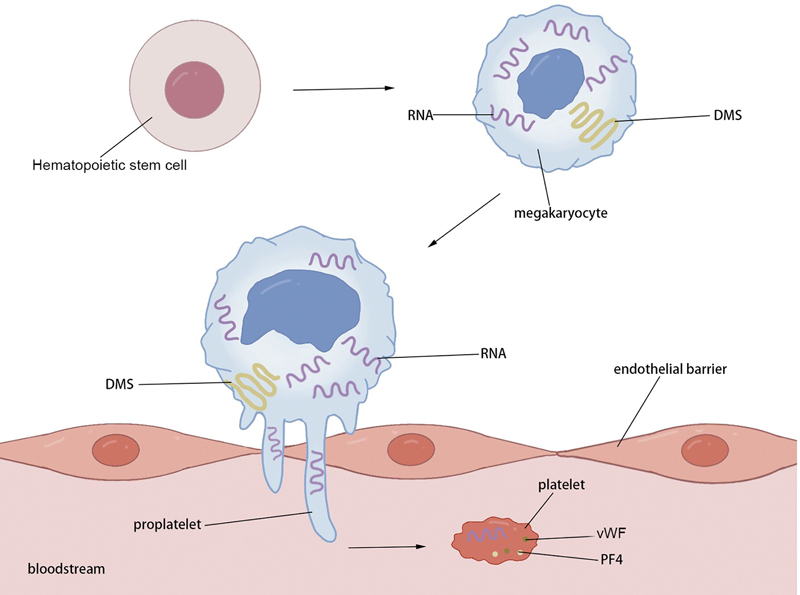
Graphical illustration of platelet formation. Hematopoietic stem cells undergo proliferation and differentiation into megakaryocytes. After the megakaryocytes mature, they extend proplatelets, which break through the endothelial barrier and mature in the blood circulation. DMS, demarcation membrane system; PF4, platelet factor 4; RNA, ribonucleic Acid; vWF, von Willebrand factor.

Platelets are mainly derived from MKs and have an average life span of only 7 to 10 days. The human body needs to produce approximately 10 ^11^ platelets per day to maintain a stable platelet count. 4 Although it is widely known that MKs are the precursor cells of platelets, the specific mechanisms and processes of platelet production and release are still not fully understood. Herein, we briefly review the known processes of platelet formation ( Fig. 1 ).

### Megakaryocyte Formation and Maturation

The traditional view is that the MK formation process begins from hematopoietic stem cells (HSCs), which continuously divide to produce multipotent progenitor cells. The degree of differentiation gradually increases during the division process, leading to the production of megakaryocyte-erythroid progenitor cells, which eventually differentiate into unipotent MK progenitor cells, and subsequently, into MK precursor cells. However, some studies identified a subpopulation of HSCs that can directly produce MK progenitor cells. 5 6 This suggests a potential pathway for the formation of MKs that skips the megakaryocyte-erythroid progenitor cell production stage, that is, direct division of HSCs to form MKs. This may represent a pathway for emergency platelet supplementation in extreme cases of platelet deficiency. However, the presence of this pathway in humans has not yet been confirmed.

MKs can perform several successive rounds of DNA replication and undergo several endomitosis to form polyploid cells. 7 In addition, MKs develop organelles, granules, and an internal membrane reservoir called the demarcation membrane system (DMS) during the continuous protein synthesis, creating conditions necessary for subsequent production of platelets. 7 Analyses conducted using focused ion beam/scanning electron microscopy revealed that the formation of the platelet plasma membrane from MK DMS involves several steps. The precursors of the DMS originate from the periphery of the cell and remain connected to the cell surface. The number of connections is related to the nuclear division status of the cell. During the development of the DMS, the Golgi apparatus assembles around the DMS and the endoplasmic reticulum transfers lipids across the DMS, eventually forming a tubular membrane network similar to the open canalicular system of platelets. 8 The DMS of the MKs provides much of the membrane structure required for platelet formation. This idea is further supported by the fact that mice with abnormal DMS show defects in platelet production. 9 However, a study by Ellis et al indicated that although mice with glycoprotein Ibα (GPIbα) mutations exhibit impaired DMS formation, proplatelet production was not affected. 10 Therefore, the production of proplatelets may involve an unclear pathway that does not depend on the membrane structure provided by the DMS.

### Proplatelet Formation and Remodeling

The first step of platelet formation after MK maturation is proplatelet extension. The structures of proplatelets and the mechanisms underlying their formation differ in vitro and in vivo. In vitro, MKs form the proplatelet extension structure in a non-polarized manner. 11 The structure is characterized by several protrusions around the cell membrane that eventually form a multi-branch tree-like extension network. 12 This process relies on microtubules and F-actin for motive force. 13 14 In vivo, the formation of proplatelet extensions by MKs is directed and polarized. 15 This is because the proplatelet extensions must be directed across the endothelial barrier to reach the blood circulation. Microtubule-binding proteins and motor proteins transport the necessary platelet particles, mitochondria, and other organelles to the slender end of the proplatelet. 7 16 Notably, the specific mechanism underlying this polarization extension is unclear; however, it may be related to the activities of F-actin and Cdc42. 17 18

Similar to the process of platelet formation in vitro, the microtubule structure plays an important role in the initiation of cytoplasmic proplatelet extension. However, once the proplatelets break through the endothelial barrier and enter the bloodstream, the microtubules have much less of an effect on extension. The elongation of proplatelets can be mediated by shear stress within the microcirculation, where the resistance of blood flow replaces the microtubule structure to stretch the proplatelet plasma membrane. 19 It has been found that the number of platelets produced by MKs cultured under microfluidic conditions is approximately 10 times that produced by MKs cultured under static conditions. 20 Notably, cultured proplatelets can mature into platelets when injected into the bloodstream. 21 These findings indicate that microcirculation remodeling is necessary for the formation of mature platelets.

The spleen and lungs may be sites of platelet production other than the bone marrow. A previous study indicated that during acute inflammation (such as sepsis), the spleen is the main site for MK and platelet production. 22 Studies conducted using microcirculation imaging revealed numerous MKs circulating in the lungs and dynamically producing platelets. 23 The pulmonary endothelial vascular microenvironment is also conducive for platelet production. 24 These sites of platelet production outside the bone marrow indicate new replenishment pathways for circulating platelet pools in vivo. However, further investigation is required to clarify the specific mechanisms underlying these pathways.

## Thrombocytopenia

Thrombocytopenia is a disease characterized by decreased platelet count in the peripheral blood. Thrombocytopenia is categorized into mild, moderate, severe, and extremely severe types based on the degree of reduction in platelet count. Generally, a platelet count of 75–100 × 10 ^9^ /L is defined as mild thrombocytopenia, 50–75 × 10 ^9^ /L as moderate thrombocytopenia, 25–50 × 10 ^9^ /L as severe thrombocytopenia, and <25 × 10 ^9^ /L as extremely severe thrombocytopenia. According to the etiologies of the disease, it can be divided into the following three categories: decreased platelet production, increased platelet consumption, and abnormal platelet distribution ( Table 1 ).

### Immune Thrombocytopenia

Immune thrombocytopenia (ITP) is an autoimmune disease characterized by low platelet count, mainly caused by increased platelet destruction and insufficient platelet production. The primary mechanism of platelet destruction in ITP is mediated by abnormal antiplatelet autoantibodies (mainly immunoglobulin G), 25 which are generally produced by abnormal B cells. Patients with ITP exhibit an increased number of splenic T follicular helper cells, which promotes the differentiation of abnormal B cells and the production of antiplatelet antibodies through the stimulation of CD40 and IL-21. 26 After binding to platelets, these autoantibodies are recognized by the FC-γ receptors on the surfaces of macrophages and mediate subsequent phagocytosis. 27 In addition to being directly involved in platelet destruction as effector cells, macrophages act as antigen-presenting cells to stimulate the proliferation of CD4 + T cells. 27 In addition, CD8 + T cells participate in platelet destruction in ITP through increased expression of perforin and granase. 28 Notably, decreased Treg cell function has been observed in patients with ITP, evidenced by decreased secretion of the Treg-derived anti-inflammatory factors IL-10 and IL-35. 29 30 In addition, patients with ITP show a decreased number of Treg cells in the spleen and bone marrow. 25 This partially explains the excessive proliferation of CD4 + , CD8 + , and abnormal B cells in patients with ITP.

Insufficient platelet production is another important cause of ITP. Thrombopoietin (TPO) is a key factor that regulates the formation of platelets from MKs and can be bound by platelets. 31 Given that the functions and quantity of MKs in patients with ITP are close to normal levels, the initial number of platelets entering the circulation is also close to the normal levels. This results in most of the TPO binding to the platelets, leading to a relatively insufficient amount of TPO regulating the production of platelets by MKs in response to a low platelet count. In addition, as MKs also express glycoprotein receptors, they are equally likely to be bound by antiplatelet receptors. Abnormal apoptosis of MKs can be observed when plasma from patients with ITP is added to cultured MKs. 25 However, whether such abnormal apoptosis is an underlying mechanism of ITP remains controversial.

### Thrombotic Thrombocytopenic Purpura

Thrombotic thrombocytopenic purpura (TTP) is an immune-mediated thrombotic microangiopathy with a high mortality rate, and is often associated with ADAMTS13 deficiency. 32 33 ADAMTS13 is an important von Willebrand factor (vWF) regulatory protein synthesized mainly in the liver, and its primary function is to cleave vWF polymers in the blood circulation and in vascular injury sites. 34 Both ADAMTS13 and vWF are secreted into the circulation in an inactive state, and the domains that can interact are exposed under the action of collagen binding and blood flow shear force. 35 The CUB domain of ADAMTS13 can interact with vWF and activate its own N-terminal MDT1CS domain. In addition, MDT1CS interacts with the A2 domain of vWF to promote the cleavage of vWF polymers. 33 35 Therefore, vWF is both a substrate and a cofactor of ADAMTS13.

Deficiency of ADAMTS13 obstructs the cleavage of vWF polymers and increases the levels of ultra-large molecular weight vWF multimers. Uncleaved vWF multimers can widely recruit platelets through the A1 domain and GPIb receptor to form transparent thrombi with platelets as the main component, leading to capillary and arteriolar embolism. 36 As platelets are retained by vWF during this process, patients often exhibit symptoms of thrombocytopenia, such as skin petechiae, ecchymoses, and mucosal bleeding. 37

The current view is that ADAMTS13 deficiency is primarily caused by genetic mutations or specific autoantibodies. ADAMTS13 mutations are diverse and cause ADAMTS13 deficiency for various reasons. A clinical study revealed 98 different ADAMTS13 mutations in 245 alleles in patients with TTP. 38 Abnormal autoantibodies are also an important cause of TTP. Polyclonal antibodies against multiple epitopes of ADAMTS13, mainly IgG, have been detected in patients with TTP. 39 These antibodies primarily target the S domain of ADAMTS13, which is critical for recognizing the A2 domain of vWF. 40 However, as some patients with TTP can remain asymptomatic for several years despite having ADAMTS13 deficiency, some scholars believe that the onset of TTP requires additional triggers, such as surgery and inflammation, to increase vWF levels. 41

### Drug-induced Thrombocytopenia

**Fig. 2:**
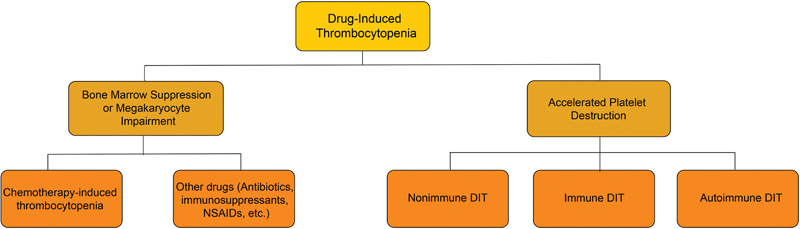
Categories of DIT. DIT can be divided into two main categories, one is myelosuppression or megakaryocyte dysfunction, and the other is excessive destruction of peripheral platelets. DIT, drug-induced thrombocytopenia; NSAID, nonsteroidal anti-inflammatory drug.

Drug-induced thrombocytopenia (DIT) is a secondary form of thrombocytopenia. The causes of DIT can be divided into two main categories: myelosuppression or MK dysfunction and excessive destruction of peripheral platelets ( Fig. 2 ). 42

Myelosuppression may be caused by drugs acting directly on MKs or their progenitors in the bone marrow or the disruption of the bone marrow microenvironment. 43 Chemotherapy-induced thrombocytopenia is the most common form of myelosuppression-related DIT, and its presentation varies widely depending on the type of cancer and the type and dose of the drug administered. 44 A retrospective study indicated that the incidence of chemotherapy-induced thrombocytopenia caused by gemcitabine is twice as high as that caused by cyclophosphamide. 44 In addition to antitumor drugs, some immunosuppressants such as tacrolimus, non-steroidal anti-inflammatory drugs such as aspirin, antibiotics such as vancomycin, antiviral agents such as interferon, and other drugs are known to have toxic effects on the bone marrow and can cause thrombocytopenia. 45 46 47 48

Owing to the excessive destruction of peripheral platelets observed in DIT, the disorder can be classified as autoimmune, immune, and nonimmune. One of the most common types of autoimmune DIT is heparin-induced thrombocytopenia. Heparin-induced thrombocytopenia is caused by an immune complex formed by the combination of heparin, platelet factor 4, and platelet factor 4 antibodies. These complexes can be recognized by platelet FcγRIIA and promote platelet activation, which leads to thrombosis. 49 This type of DIT is often mediated by drug-dependent antibodies. Hundreds of drugs that may cause immune-related DIT are listed on a Web site called “Platelets on the Web” ( https://www.ouhsc.edu/platelets/ditp.html ). Non-immune-related DIT can occur in thrombotic microangiopathy and some hemolytic uremic syndromes, mainly because of the direct toxic effects of some chemotherapy drugs and immunosuppressive drugs on platelets (such as cyclosporine and tacrolimus). 43

## Medication Strategies for the Treatment of Thrombocytopenia

### Corticosteroids

Considering the abovementioned pathological mechanisms of thrombocytopenia, high-dose corticosteroid shock suppression of abnormal immune responses remains the first-line treatment for ITP. 50 Corticosteroids increase platelet production, decrease platelet clearance, and act on blood vessels to reduce bleeding. 50 The most commonly used treatment options include pulsed high-dose dexamethasone or prednisone therapy. Notably, there is no statistically significant difference between the effects of these therapies at 6 months post-therapy. 51 The initial response rate for dexamethasone is approximately 60 to 80%; however, only 20 to 40% of patients maintain a response after discontinuing the drug. 52 In addition, as cortisol has long-term adverse reactions, the benefits of corticosteroid treatment are fewer than its side effects. 53 Therefore, long-term use of corticosteroids is not recommended.

Corticosteroids can be used as adjuncts to therapeutic plasma exchange (TPE) for the treatment of patients with TTP. Despite the limited amount of relevant research on treatment options for TTP, patients with TTP are often treated using empirical corticosteroid treatment (1.5 mg/kg/day for 3 consecutive weeks). 54 Notably, higher doses of corticosteroids show better therapeutic effects. 55 However, there is still a lack of evidence that corticosteroids have clear benefits for the treatment of TTP.

### Intravenous Immunoglobulin

Intravenous immunoglobulin (IVIg) is another commonly used first-line treatment for newly diagnosed ITP. The response rate for the treatment is up to approximately 80% in newly treated patients; however, the response is usually short-lived. 56 As IVIg is relatively established as a first-line drug for the treatment of ITP and its price is relatively high, recent studies on IVIg were mostly focused on the optimization of IVIg infusion and reducing the burden of treatment. The therapeutic dose of IVIg is usually 1 g/kg/day for 1 to 2 days or 0.4 g/kg/day for 5 days. The total dose and efficacy of these regimens are similar. 57 A clinical study by Zhou et al indicated that the efficacy of a low dose (0.2 or 0.3 g/kg/day) of IVIg is similar to that of a conventional dose (0.4 g/kg/day). 58 In addition, the efficacy and adverse event rates of 10% IVIg are similar to those of 5% IVIg. 50 59 60 Therefore, appropriate reduction of the IVIg dose can be an economical and effective treatment option for patients with ITP. In addition to considering the risk of renal insufficiency and thrombosis, the presence of anti-platelet antibodies should be considered before administration of IVIg. 61 Some studies have shown that GPIb-IX antibody-positive patients with ITP show a much lower response to IVIg treatment than GPIb-IX antibody-negative patients (36.4% versus 80%). 62 However, as most patients have multiple antibodies, this conclusion remains controversial.

### Thrombopoietin Receptor Agonists

Thrombopoietin receptor agonists (TPO-RAs) are a class of drugs that promote platelet production. They can act directly on TPO receptors on the surface of MKs to promote differentiation and development of MKs and platelet formation. TPO-RAs are mainly used for long-term treatment of chronic ITP that does not show sufficient response to the initial treatment. They are also used for the treatment of thrombocytopenia caused by chronic liver disease, aplastic anemia, and treatment after hematopoietic stem cell transplantation. 63 The Food and Drug Administration (FDA) and European Medicines Agency (EMA) have approved three TPO-RAs for the treatment of persistent or chronic ITP: eltrombopag, romiplostim, and avatrombopag.

Eltrombopag is a small-molecule non-peptide TPO-RA. The initial dose for routine dosage is 25 or 50 mg/day, and the maximum dose is 75 mg/day. However, the specific dose administered depends on certain factors such as the patient's age, race, and liver function. 64 Eltrombopag is a relatively established TPO-RA and has been studied extensively for the treatment of chronic ITP. Although the reported response rates for eltrombopag vary across studies, ranging from approximately 50 to 90%, the reported rates were all statistically different from those observed in placebo groups. In addition, patients who received eltrombopag showed significantly reduced bleeding rates and incidences of emergency events that required rescue. 65 66 67 However, it should be noted that eltrombopag can chelate metal ions; therefore, oral administration should be performed at least 2 hours before or 4 hours after taking drugs or foods that contain polyvalent cations. 56 68 If no response is observed after 4 weeks of treatment with the maximum dose, discontinuation of the therapy is recommended. 50

Avatrombopag is another TPO-RA that was approved recently for the treatment of chronic ITP. Avatrombopag is similar to eltrombopag and is a small-molecule, non-peptide TPO-RA that acts on TPO receptors without competing with TPO. 63 The starting dose of avatrombopag is usually 20 mg/day, and the maximum dose is 40 mg/day, depending on the patient's case. In a phase II clinical trial, approximately 93% of patients with ITP showed increased platelet count of up to >50 × 109/L on the seventh day after taking avatrombopag 20 mg/day, whereas the placebo group showed only a 7% increase in platelet count. 69 In another phase III study, the median cumulative time to recovery of platelet count of up to >50 × 109/L in the avatrombopag treatment group was 12.4 weeks, whereas that in the placebo group was 0 week. 70 Unlike eltrombopag, avatrombopag does not chelate metal ions, interact with drugs or food, or require monitoring of liver function. In addition, it is better absorbed with food. 71

Romigrastim differs from the two abovementioned drugs in that it is the only TPO-RA with an action site that is the same as that of endogenous TPO. 72 The usual starting dose of romiplostim is 1 or 3 μg/kg/week subcutaneously, which can be titrated up to a maximum of 10 μg/kg/week. Several clinical studies have shown that romigrastim can significantly increase and maintain platelet counts, with response rates ranging from approximately 74 to 96%. 73 74 75 If the maximum dose of 10 μg/kg/week is not effective after 4 weeks of treatment, discontinuation of romiplostim is recommended. 50 In addition, if the treatment response rate suddenly decreases, the possibility that antibodies against romigrastim have been formed should be considered.

### Rituximab

Rituximab is a monoclonal antibody that targets B cells expressing CD20. It is typically used for the treatment of lymphomas and some immune system diseases. However, it is also widely used for the treatment of ITP and TTP. Long-term data on rituximab treatment for ITP indicates that rituximab effectively improves the platelet count of patients at 6 months but does not improve the risk of bleeding. 76 The usual therapeutic dose of rituximab for ITP is 375 mg/m ^2^ /week for 4 weeks, and the response rate at this dose ranges from approximately 60 to 80%. Notably, the response rate may be affected by factors such as the course of the disease, sex, and age. 77 78 79 80 Another dosing regimen (1000 mg/m ^2^ on days 1 and 15, 100 mg/m ^2^ per week for 4 consecutive weeks) has shown similar short-term efficacy, 81 82 with an overall remission rate of >50%. A major problem with rituximab treatment is the high relapse rate, which is caused by the consumption of anti-CD20 antibodies and gradual recovery of B cells. A study by Deshayes et al indicated that the relapse rates after rituximab treatment are 38, 31, and 21% at 1, 2, and 5 years, respectively. 81

Regarding the treatment of TTP, rituximab is mainly used for patients who do not respond well to conventional treatments. The usual treatment dose is the same as that for ITP (375 mg/m ^2^ per week for 4 consecutive weeks). 54 Rituximab has shown good results in several small-scale studies on the treatment of TTP, with a short-term overall remission rate of approximately 90%. 83 84 85 In addition, patients with TTP treated with rituximab seem to have fewer and later relapses. 83 These data indicate that rituximab has the potential to become a first-line treatment for TTP.

### Fostamatinib

Fostamatinib is the first commercialized splenic tyrosine kinase inhibitor that mainly inhibits splenic tyrosine kinase in vivo, blocks Fc and B cell receptor signaling, and reduces the recognition and degradation of platelets by the immune system. It was approved by the FDA and EMA for the treatment of ITP in 2018. The initial treatment dose for fostamatinib is 100 mg twice a day, and the dose can be increased to 150 mg for patients who show insufficient response to treatment. Two phase III clinical trials on the use of fostamatinib for the treatment of ITP indicated that the overall response rate at 100 mg twice a day was 43% in the fostamatinib group and 14% in the placebo group. 86 However, the stable response rate was only 18% in the treatment group and 2% in the placebo group. 86 At the subsequent long-term follow-up, approximately half of the patients achieved an overall treatment response, and most maintained it for more than 2 years. 87 If no response is observed after 12 weeks of treatment with fostamatinib, discontinuation of the drug is recommended. 50

### Therapeutic Plasma Exchange

TPE is the most effective treatment option for TTP. It can not only remove autoantibodies in the circulation but also replenish ADAMTS13 in the plasma. As the mortality rate of untreated TTP is extremely high, TPE should be performed immediately after a diagnosis of TTP is established or highly suspected. 54 TPE should be performed every day until the platelet count is restored to normal levels and organ involvement is significantly relieved. TPE is primarily performed through the central venous channel. However, peripheral venous channels can also be used if the central venous channel is affected by lesions or other conditions. The usual replacement volume for TPE is 40 mL/kg; however, 60 mL/kg can be administered initially at least once a day. 37 Patients with severe conditions or neurological symptoms can undergo the procedure more than once a day. 88 However, if the optimal conditions for TPE cannot be achieved, plasma infusion is recommended as a temporary treatment. 89

### Caplacizumab

Caplacizumab is a new nanoantibody drug that targets vWF. Caplacizumab was approved by the EMA and FDA in 2018 and 2019, respectively, specifically for the treatment of adult-acquired TTP. Caplacizumab can block the interaction between vWF and platelets, thereby reducing platelet adhesion and excessive consumption mediated by vWF. 90 91 Its usual dosing regimen is 10 mg intravenously before plasma exchange on the first day of treatment, followed by 10 mg subcutaneously every day after plasma exchange for 30 days. A double-blind controlled clinical trial revealed that patients who received caplacizumab showed a significantly shorter median time to recovery of platelet counts than those who received placebo treatment. In addition, the likelihood of treatment remission in the caplacizumab group was 1.55 times higher than that in the placebo group. 90 Moreover, the TTP recurrence rate was only 12% in the caplacizumab group and 38% in the placebo group. 90 The authors of another study proposed an optimal dosing regimen of alternate-day dosing. 92 In their study, most patients treated using the alternate-day dosing regimen maintained normal platelet counts; however, five patients still showed deterioration or relapse, leading to re-initiation of daily dosing. The findings of the study suggest that if ADAMTS13 activity remains <10% after 3 to 4 weeks of daily treatment, alternate-day dosing can be considered; however, platelet counts must be strictly monitored and thrombotic microangiopathy must be observed. 92

### Splenectomy

As the spleen is the primary site of platelet clearance and is associated with platelet antibody production, splenectomy is a treatment option for ITP. Although splenectomy for ITP is rarely performed at present, a 2019 expert consensus on the treatment of ITP still listed splenectomy as a second-line treatment for ITP, along with administration of TPO-RAs, rituximab, and fostamatinib. 50 The consensus recommended that splenectomy be performed 1 to 2 years after diagnosis and that platelet counts be increased to >50 × 10 ^9^ as much as possible before surgery. Several clinical studies have shown that the response rate to splenectomy is approximately 80 to 90%. 93 94 95 However, as many patients often undergo the surgery while on other treatments, this percentage may be exaggerated. In addition, it should be noted that the risks of thromboembolism and infection significantly increase after splenectomy. 96

The use of TPO-RAs, rituximab, and other drugs has significantly improved the management of ITP. Although most current guidelines still recommend splenectomy as the second-line treatment for ITP, clinicians often prefer to delay or avoid performing splenectomy because of its expected efficacy, associated complications, or low patient willingness. However, splenectomy remains an effective treatment option for some patients with ITP who show no response to drug therapy and have no comorbidities. 97

## Thrombocytosis

Thrombocytosis is a platelet disorder characterized by high platelet count, A peripheral platelet count ≥450 × 10 ^9^ is typically used as the diagnostic cut-off for thrombocytosis. The most common types of thrombocytosis are reactive thrombocytosis and essential thrombocythemia (ET).

### Reactive Thrombocytosis

Reactive thrombocytosis is the most common type of thrombocytosis, and is usually observed in various types of infections, inflammatory states, after surgery, and in other traumatic processes. 98 99 Reactive thrombocytosis is typically driven by TPO and various cytokines, 100 and patients often exhibit significantly elevated IL-6 and C-reactive protein levels, which can be used to determine whether the thrombocytosis is clonal or reactive. 101 As reactive thrombocytosis is a secondary disease, it can often be controlled better after active treatment of the primary disease. However, the direct risk of increased platelet count should be evaluated. In addition, the risks of thrombosis and other complications should be considered.

### Essential Thrombocythemia

ET is a myeloproliferative neoplasm mainly driven by mutations of *JAK2* (janus kinase 2), *CALR* (calreticulin), and *MPL* (myeloproliferative leukemia virus oncogene). The *JAK2* mutation is the most common, expressed in approximately 50 to 60% of patients with ET. 102 103 *CALR* and *MPL* mutations are present in approximately 20 to 25% and 3 to 4% of patients, respectively. 104 However, these three driver mutations, known as triple-negative ET, are not present in approximately 10 to 15% of patients with ET. 105 In addition, a small number of patients with ET will develop myelofibrosis, known as post-ET MF, as the disease progresses.

*JAK2* mutations promote clonal bone marrow proliferation, including extramedullary hematopoiesis and MK proliferation, by directly activating JAK2 and signal transducers and activators of transcription (JAK2-STAT). 106 Mutant *CALR* secretes abnormal CALR proteins, which specifically bind with MPL on MKs and play a role in promoting platelet formation, a function similar to that of TPO. 107 In addition, patients with ET who have *MPL* mutations appear to have a higher likelihood of developing myelofibrosis than those with other mutations. 108 In a previous study, patients with ET were compared (regardless of mutation status, including triple-negative ET) with healthy volunteers through pathway analysis. The results revealed that the patients with ET showed significant upregulation of tumor necrosis factor, mitogen-activated protein kinase, and nuclear factor kappa-B pathways. 105 This suggests that ET phenotypes, regardless of the mutation type, may be caused, at least in part, by transcriptional misregulation.

## Medication Strategies for Thrombocytosis

### Interferon

Interferon (IFN) is a cytokine with antiviral, anti–cell proliferation, antitumor, and immunomodulatory functions. However, IFN has many side effects; therefore, it is not commonly used for the treatment of ET. The emergence of pegylated IFN (peg-IFN) has allowed the use of an IFN as a first-line treatment for ET. The pharmacokinetics of peg-IFN are different from those of standard IFN. In addition, peg-IFN supports a lower dosing frequency and can therefore improve patient tolerance. 109 Studies have shown that peg-IFN is more selective for HSCs than standard IFN. 110 The usual dosage of peg-IFN is approximately 90 μg per week subcutaneously, which is associated with an overall remission rate of approximately 80% for the treatment of ET and a reduction in the allele fractions of the *JAK2* V617F variant. 111 112 A meta-analysis on the efficacy and adverse effects of peg-IFN and non-peg-IFN for the treatment of ET indicated that both types of IFN have similar overall response rates (79.8 and 80.8%, respectively). 113 The most common adverse effect of IFN therapy is flu-like symptoms, which are more common with the use of non-Peg-IFN. 113 Notably, IFN treatment for ET is associated with an overall response rate not less than that associated with hydroxyurea. 114 Moreover, genomic analysis has shown that patients receiving IFN exhibit considerable decrease in allele fractions of the *JAK2* V617F variant. 115 Although peg-IFN reduces the risk of adverse reactions while ensuring good therapeutic effects, the incidence of adverse reactions following peg-IFN therapy is still high. In addition, it is not available in oral preparations. Therefore, further research is needed to determine other considerations for its clinical applications.

### Aspirin

Aspirin, a non-steroidal anti-inflammatory drug, inhibits cyclooxygenase-1, which leads to inhibition of the release of thromboxane A2, platelet activation, and thrombosis. Studies have demonstrated that patients with ET show increased levels of thromboxane A2 metabolites, which is related to the degree of increase in platelet count. 116 In addition, many patients with ET show significantly elevated platelet aggregation levels. 117 A conventional preventive aspirin dose of 81 to 100 mg/day is recommended for the prevention of thrombosis in patients with ET. Notably, a study revealed that in patients with ET who are younger than 60 years and have no history of thrombosis, aspirin can reduce the incidence of venous thrombosis and arterial thrombosis in those with *JAK2* mutations but will increase the incidence of bleeding events in those with platelet counts >1000 × 10 ^9^ . 118 Another study showed similar experimental results, that is, aspirin has a preventive effect on thrombosis only in patients with *JAK2* mutations but increases the risk of bleeding. 119 Therefore, although low-dose aspirin is the recommended therapy for preventing thrombotic events in all patients with ET, observation should be preferred for low-risk patients with platelet counts >1000 × 10 ^9^ or *CALR* mutations, whereas cytoreductive surgery rather than aspirin should be preferred for high-risk patients with platelet counts >1000 × 10 ^9^ or *CALR* mutations. 117

### Lysine-specific Demethylase 1 Inhibitor

Lysine-specific demethylase 1 (LSD1) was the first histone demethylase to be identified. LSD1 regulates cell transcription by demethylating mono- or dimethyl groups on histone H3. Pathologically, LSD1 is involved in the development and metastasis of a variety of tumors, including small cell lung cancer, prostate cancer, breast cancer, and some hematological tumors. 120 In the hematopoietic system, LSD1 knockdown can promote the proliferation of HSCs and MKs but severely inhibit the production of terminal granulocytes, red blood cells, and platelets. 121 Bomedemstat is a small-molecule LSD1 inhibitor used for the treatment of myelofibrosis, ET, and polycythemia vera. In a multicenter clinical trial on the efficacy and safety of bomedemstat for the treatment of ET, all 29 patients treated with bomedemstat for more than 6 weeks showed platelet count reduction, with 90% (26/29) of the patients achieving platelet counts ≤400 × 10 ^9^ /L and no thromboembolic events. 122 In a phase II clinical trial on the use of bomedemstat for ET, 94% (34/36) of the patients treated for more than 24 weeks achieved platelet counts of ≤400 × 10 ^9^ /L within a median of 8 weeks. In addition, 83% (30/36) of the patients maintained their treatment response for more than 12 weeks with no new thromboembolic events. 122 Although bomedemstat is still not widely used, recent clinical trial results indicate that it has excellent clinical application prospects.

### Therapeutic Thrombocytapheresis

Therapeutic thrombocytapheresis is a clinical treatment in which the blood of the patient is collected and the pathological components are separated, removed, or replaced by setting the relevant procedures in the blood cell separator. As patients do not respond to medications immediately, therapeutic thrombocytapheresis is often the first choice for rapid platelet clearance in patients with severe thrombocytosis and/or thrombohemorrhagic complications. 123 In single-center retrospective study of 185 patients with myeloproliferative neoplasms and thrombocytopenia who underwent therapeutic thrombocytapheresis, the median proportion of patients with thrombocytopenia was 44.5%, the median removal response rate was 65.2%, and adverse events were rare (13/185). 123 These findings indicate that therapeutic thrombocytopheresis is an effective treatment option for thrombocytopenia. In clinical practice, combination of medications with therapeutic thrombocytapheresis can be considered depending on the degree of thrombocytosis observed and the patient's risk of complications.

### Shear Stress-related Platelet Dysfunction

Cardiovascular therapeutic devices (CTDs), such as mechanical circulatory support (MCS) and extracorporeal membrane oxygenation (ECMO), are important treatments for patients with heart failure. However, although these devices provide hemodynamic support for patients, the shear stress generated in the blood flow also has an impact on platelet activation, which clinically manifests as coagulation system disorders characterized by the coexistence of thrombosis and bleeding events. 124

Device-related thrombosis begins with shear-mediated platelet activation (SMPA). A study found that platelet activation caused by mechanical stress produces different biomarkers from those mediated by agonists. 125 Under conditions of continuous shear force simulating CTDs without agonists, platelet phosphatidylserine externalization (PSE) levels and procoagulant activity increase significantly. However, the expression of the traditional platelet activation biomarker P-selectin does not change significantly. 125 Another study proposed a potential mechanism of SMPA: shear stress increases platelet membrane damage, leading to activation of platelet shear-sensitive pores and channels, allowing specific agonists to enter the cell and promote platelet activation. 126

CTD-related bleeding events are also serious complications that threaten patients, but we still know little about the specific mechanism. Studies have found that when platelets are exposed to high shear forces, the expression of platelet-derived microparticles rich in platelet receptors increases significantly. 127 128 This causes the main adhesion receptors GPIbα, GPVI, and GPIIb/IIIa to fall off the platelet surface along with the platelet-derived microparticles, 129 130 thereby limiting the activation and aggregation of platelets. This partly explains the bleeding tendency of patients using CTDs.

### Medication Strategies for Shear Stress-related Platelet Dysfunction

Patients treated with CTDs are often at risk of developing thrombosis and bleeding at the same time. Because thrombosis and bleeding caused by shear stress cannot be distinguished from other conditions in clinical practice, there is currently a lack of corresponding specific medication. In cases of thrombosis or suspected thrombosis, drugs such as unclassified heparin, antithrombin, and urokinase are commonly administered. 131 When bleeding occurs, the source of bleeding should be identified first and local treatment should be actively provided. At the same time, the cause should be identified, and drugs such as blood products, protamine, and vitamin K should be administered, depending on the cause. 124 131

## Summary

The importance of platelets in the hemostatic and immune systems has been confirmed in numerous studies. Although we have a preliminary understanding of the regulation of platelet production and related diseases, several pathophysiological mechanisms underlying the development of platelet disorders need to be explored further. In this review, we summarized the currently known mainstream mechanisms underlying the regulation of platelet production and the factors associated with them. In addition, we reviewed some common platelet-related diseases and common drug strategies used for their treatment ( Table 2 ).

**Table 2:** Summary of medications discussed in this research

Medication		Indications (discussed in this research)	Conventional dose
Corticosteroid		ITP and adjuvant therapy for TTP	Depend on clinical
Intravenous immunoglobulin		ITP	1 g/kg/day for 1–2 days or 0.4 g/kg/day for 5 days
Thrombopoietin receptor agonists	Eltrombopag	Chronic ITP	25 or 50 mg/day
	Avatrombopag		20 mg/day
	Romigrastim		1 or 3 μg/kg/week
Rituximab		ITP and TTP	375 mg/m ^2^ /week for 4 weeks
Fostamatinib		ITP	100 mg twice a day
Therapeutic plasma exchange		TTP	40 mL/kg (replacement volume)
Capecizumab		TTP	10 mg/day after plasma exchange for 30 days
Splenectomy		ITP	N/A
Pegylated interferon		ET	90 μg/week
Aspirin		ET (prevention of thrombosis)	81–100 mg/day
Inhibitor of lysine-specific demethylase 1	Bomedemstat	ET	Not clear
Therapeutic thrombocytapheresis		ET	N/A

Abbreviations: ET, essential thrombocythemia; ITP, immune thrombocytopenia; N/A, not applicable; TTP, thrombotic thrombocytopenic purpura.

Given that platelet production and regulation are complex and variable, there is still an unmet need for comprehensive research on the diagnosis and treatment of platelet-related diseases to allow for more accurate diagnoses and the development of more individualized treatment regimens. We believe that this review will serve as a reference tool for further research on platelet disorders, which could drive better understanding of clinical manifestations, immune characteristics, and genetic factors to optimize the treatment of platelet disorders in clinical practice.
